# Activation of Ang-(1-7)/Mas Receptor Is a Possible Strategy to Treat Coronavirus (SARS-CoV-2) Infection

**DOI:** 10.3389/fphys.2020.00730

**Published:** 2020-06-19

**Authors:** Giselle Santos Magalhaes, Maria da Gloria Rodrigues-Machado, Daisy Motta-Santos, Maria Jose Campagnole-Santos, Robson A. Souza Santos

**Affiliations:** ^1^Department of Physiology and Biophysics, National Institute of Science and Technology in Nanobiopharmaceutics (INCT-Nanobiofar), Belo Horizonte, Brazil; ^2^Medical Sciences Faculty of Minas Gerais, Post-graduate Program in Health Sciences, Belo Horizonte, Brazil

**Keywords:** corona virus, angiotensin-(1-7), angiotensin II, ACE2, SARS-CoV-2, lung, endothelium

## Introduction

Coronavirus disease (COVID)-19 is developed from the virus entering the host cells, initially in the airways, mouth, eyes, and lungs. The cell membrane of these tissues expresses, with different densities, one or more proteins that allow the binding of the “severe acute respiratory syndrome corona virus 2” (SARS-CoV-2) spike protein and its entry into the cell. Angiotensin converting enzyme 2 (ACE2, Kuba et al., 2005; Wang et al., 2020), transmembrane serine protease 2 (TMPRSS2; Matsuyama et al., 2020), sialic acid receptors (Hulswit et al., 2019; Tortorici et al., 2019), and extracellular matrix metalloproteinase inducer (CD147; Chen et al., 2005) have been demonstrated as possible binding proteins for SARS-CoV-2. Among these, ACE2 is largely expressed in airway cells, in alveolar epithelial type II cells and in endothelial cells (Donoghue et al., 2000; Santos et al., 2018; Xu et al., 2020). The disease starts with pulmonary symptoms with high deficit of blood oxygenation and indication of pneumonia and the worsening of the disease clearly indicates major impairment of the vascular endothelium, i.e., high blood pressure, thrombosis (Zhang et al., 2020; Zhou et al., 2020), pulmonary thromboembolism (Bikdeli et al., 2020; Rotzinger et al., 2020), stroke and myocardial infarct (Aggarwal et al., 2020; Klok et al., 2020). In fact, diffuse pulmonary endothelial cell injury, that results in impairment of the alveolar–capillary barrier and increase in microvascular endothelial permeability, is considered central to the pathogenesis of acute respiratory distress syndrome (ARDS; Cheng et al., 2007).

The ACE2 removal from the cell membrane due to SARS-CoV-2 binding is an important factor for the worsening of the disease. ACE2 is a key enzyme of the renin-angiotensin system (RAS), that converts with high affinity angiotensin (Ang) II to Ang-(1-7) (Rice et al., 2004; Santos et al., 2018). Reduction in ACE2 cell membrane availability will alter the balance of the RAS toward an increase in Ang II and a decrease in Ang-(1-7) in the lungs, in the circulation, in the vessels, virtually in all organs with few exceptions (Liu et al., 2020). Experimental and clinical evidences indicate that activation of Ang-(1-7)/Mas receptor is an important mechanism to fight the deleterious effects triggered by an inappropriate increase in Ang II/AT_1_ receptor in different diseases (Santos et al., 2018). Thus, activation of the Mas receptor or administration of Ang-(1-7) or Mas analogs can be important additive measures to control the inflammatory response mediated by SARS-CoV-2, as already pointed out by Peiró and Moncada (2020) and Shete (2020).

## ACE2 and Acute Lung Diseases

In ARDS, it has been demonstrated an imbalance between ACE and ACE2 activity favoring ACE activity, which correlated with higher degree of lung injury (Wang et al., 2019). Further, Imai et al. (2005) reported that lack of ACE2 expression (knockout mice, ACE2^−/Y^) precipitated severe ARDS, suggesting that ACE2 could have an important role in mitigating ARDS. In this study, elastance of the respiratory system and pulmonary edema were significantly higher in ACE2^−/Y^ mice subjected to a model of sepsis. In addition, it was observed thickening of the alveolar wall, pulmonary congestion and edema, infiltration of inflammatory cells, and hyaline membrane in these mice. After 6 h of observation, all WT animals were alive and only 2 out of 10 animals in the ACE2^−/Y^ group survived. Moreover, intraperitoneal injection of recombinant human ACE2 protein (rhuACE2) in ACE2^−/Y^ mice subjected to ARDS prevented the increase in respiratory system elastance and pulmonary edema. In contrast, ACE knockout animals (ACE^−/−^) were protected against ARDS induced by acid aspiration and ACE inactivation in ACE2^−/Y^ animals attenuated ARDS. Likewise, pharmacological inhibition or genetic deletion of AT1a (AgTr1a^−/−^) receptors significantly attenuated pulmonary dysfunction and edema (Imai et al., 2005). It is interesting to note that ACE inhibition or blockade of AT1 receptor favors an increase in Ang-(1-7) levels in rats and humans (Kohara et al., 1993; Santos et al., 2018). However, if AT1 blockade is associated with a decrease in ACE2 availability, such as in SARS-CoV-2 infection, the production of Ang-(1-7) will be compromised and thus, part of the beneficial effects can be lost.

Bone marrow-derived mesenchymal stem cells (MSCs) with ACE2 overexpression were used as a vehicle for gene therapy in ARDS mice induced by lipopolysaccharide (LPS; He et al., 2015). In animals that received these cells, pulmonary overexpression of ACE2 was associated with improved lung histopathology, decreased neutrophils, and inflammatory mediators in the lung, decreased Ang II and increased ACE2 and Ang-(1-7) pulmonary levels (He et al., 2015). In addition, attenuation of lung inflammatory response and tissue injury induced by pulmonary ACE2 overexpression was abolished by an ACE2 inhibitor (Li et al., 2016). On the other hand, ACE2 knockdown caused a marked deterioration of lung injury and increased cytokine secretion in rats that received LPS injection (Li et al., 2016). Altogether, these findings suggest that ACE2 in the lung prevents LPS-induced lung inflammation and injury. Moreover, it has been shown that pretreatment with either A779, a selective antagonist of Ang-(1-7) receptor, Mas, or an ACE2 inhibitor significantly inhibited the protective effects of ACE2 on LPS-induced lung injury (Li et al., 2016). This observation indicates that the protective action of ACE2 on lung injury in ARDS is mediated by Ang-(1-7).

## Angiotensin-(1-7) and the Immune System

Several studies have shown that treatment with Ang-(1-7) or other Mas receptor agonist activates an anti-inflammatory response in different pathophysiological conditions (Rodrigues-Prestes et al., 2017; Santos et al., 2018). More recently, we have also described that pro-resolving mechanisms are triggered by Ang-(1-7) in acute and chronic inflammatory processes (Barroso et al., 2017; Magalhaes et al., 2018). In the lung, Mas receptor is expressed in the epithelium and airway smooth muscle, alveolar cells, vascular smooth muscle cells, and endothelium (Wösten-van Asperen et al., 2011; Magalhaes et al., 2015). It has also been identified in cells of the immune system, such as, dendritic cells, lymphocytes, macrophages, eosinophils, neutrophils, and alveolar macrophages, indicating a cellular mechanism for immune actions by Ang-(1-7) (Barroso et al., 2017; Rodrigues-Prestes et al., 2017; Magalhaes et al., 2018; Santos et al., 2018).

In models of pulmonary inflammation, such as asthma, lung fibrosis, ARDS, and pulmonary emphysema, administration of Ang-(1-7) decreased cytokine/chemokine synthesis, migration of inflammatory cells to the lung and improved pulmonary function (Shenoy et al., 2010; Chen et al., 2013; Klein et al., 2013; Magalhaes et al., 2015, 2018; Zambelli et al., 2015; Rodrigues-Prestes et al., 2017; Santos et al., 2018; Bastos et al., 2019). Ang-(1-7) treatment improved arterial oxygenation, decreased inflammatory response, and reduced collagen deposition in the lungs of murine ARDS models (Chen et al., 2013; Zambelli et al., 2015) suggesting that the inhibitory effect of Ang-(1-7) in the recruitment of inflammatory cells observed in the acute phase may be related to the reduction in fibrosis in the later phase. In addition, anti-inflammatory effects were also observed after treatments with the ACE inhibitor, captopril, and/or the receptor antagonist, losartan (Jerng et al., 2007; Jiang et al., 2007). The effects of these treatments may also be partially related to an increase in Ang-(1-7) levels in the lung (Kohara et al., 1993; Santos et al., 2018). In another example of chronic lung inflammation, such as asthma, treatment with Ang-(1-7) was shown to reduce eosinophils count in the lung, to reduce the production of inflammatory mediators, to decrease activation of signaling pathways related to the production of cytokines, chemokines, and survival of inflammatory cells (Magalhaes et al., 2015, 2018). These anti-inflammatory effects were accompanied by reduced collagen deposition, mucus production, and improved lung function (Magalhaes et al., 2015, 2018).

An important step in the immune response aimed to restore tissue homeostasis is resolution of inflammation. We have recently demonstrated that Ang-(1-7) is a pro-resolutive mediator (Barroso et al., 2017; Magalhaes et al., 2018). We found that treatment with Ang-(1-7) at the peak of pulmonary eosinophilic inflammation induced eosinophil apoptosis, inhibited signaling pathways related to cytokine production and inflammatory cell survival, and reduced molecules related to maintenance of the Th2 immune response (Magalhaes et al., 2018). In addition, Ang-(1-7) reduced the expression of genes involved in collagen expression in the lung (Magalhaes et al., 2018). We have also observed similar results in a model of neutrophil-induced inflammation, arthritis (Barroso et al., 2017). In this condition, blocking the Mas receptor delayed natural resolution, emphasizing that the ACE2/Ang-(1-7)/Mas axis plays an important physiological role in the resolution of inflammation (Barroso et al., 2017). Moreover, in FVBN mice, a strain resistant to the asthma model, genetic deletion of Mas receptor worsened pulmonary inflammation and remodeling when these animals were subjected to ovalbumin-induced asthma (Magalhaes et al., 2016). Therefore, in addition to the therapeutic administration of Ang-(1-7), results from our group strongly suggest that absence or malfunction of the ACE2/Ang-(1–7)/Mas pathway intensifies inflammation, affects its resolution, and contributes to the impaired function of the inflamed tissue.

It is now becoming clearer that COVID-19 is mainly a result of a diffuse endothelial cell injury. Initially the virus reach the lung, and patients evolve with important deficit of blood oxygenation probably due to thickening of lung parenchyma, vascular damage, and pulmonary edema associated to disseminated intravascular coagulation. The endothelium is rich in ACE2 and its removal from the cell membrane, as the virus enters the cell, reduces its enzymatic function also at the vessel wall, generating RAS imbalance with the deleterious result of inflammation, fibrosis, and thrombosis.

## Conclusion: the Way Forward

**Figure 1:**
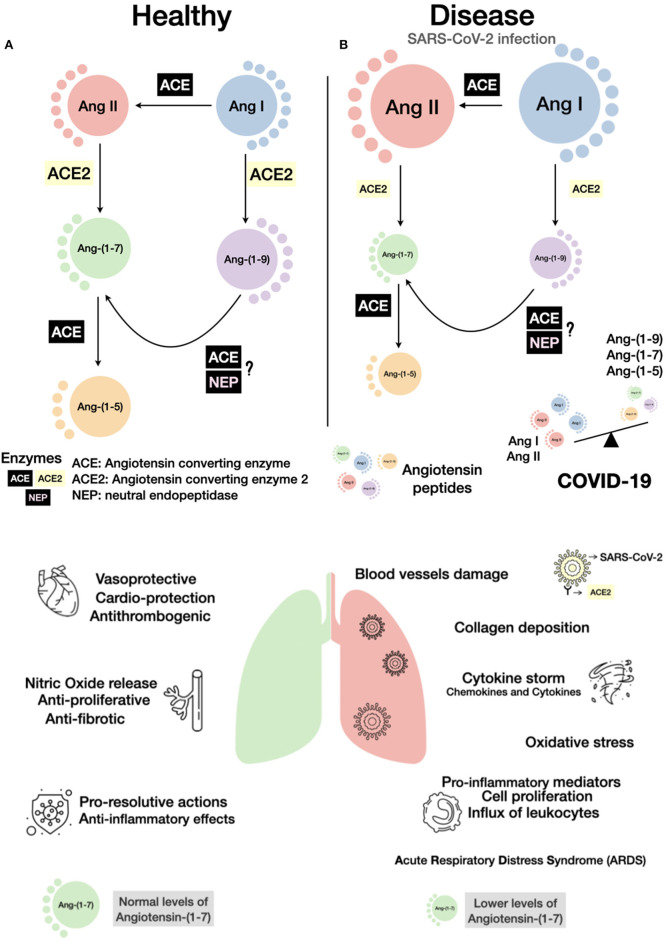
Schematic view of the consequences of COVID-19 on the renin-angiotensin system (RAS). A simplified RAS cascade with the main peptides and enzymes is shown. The size of the circles represents possible amounts of the peptides in a healthy **(A)** or disease condition, such as in SARS-CoV-2 infection **(B)**. As ACE2 is internalized by SARS-CoV-2 binding, its hydrolytic capacity in reduced, an imbalance of the RAS peptides can be established with a rise of Ang II and a decrease in Ang-(1-7) levels. Bellow enzymatic cascades, the main effects induced by normal (left) or altered (right) balance of Ang II/Ang-(1-7) are presented. Considering the anti-inflammatory and pro-resolutive effects of Ang-(1-7), activation of the Mas receptor or administration of Ang-(1-7) or analogs can be important additive pharmacological measures to control COVID-19.

There is mounting evidence that the protective arm of the RAS plays a central role in many functions linked to this system. The heart, lungs, brain, and blood vessels are among the organs in which the ACE2 product, Ang-(1-7) exerts actions that include cardioprotection, improvement of endothelial function, beneficial central actions (stroke, baroreflex, blood pressure, stress coping behaviors), anti-thrombogenic, anti-inflammatory, and pro-resolving effects (Figure 1; Santos et al., 2018). In terms of inflammation, Ang-(1-7) appears to play a crucial role in many organs including: joints, brain, lung, and kidney not only by its anti-inflammatory, but also for its pro-resolutive and anti-remodeling actions (Barroso et al., 2017; Rodrigues-Prestes et al., 2017; Magalhaes et al., 2018). On the contrary, the ACE2 substrate Ang II exerts opposing effects to those of Ang-(1-7). The devastating effect of the SARS-CoV-2 virus in the body appears to rest in part in the acute imbalance of the local and systemic concentration of these counteracting peptides. By binding to ACE2 and sequestering it from the cell membrane, the SARS-CoV-2 may amplify its pro-inflammatory effect. Although measurements of these peptides during the Covid-19 are scarce, the available data are in accordance with this possibility (Liu et al., 2020). Indeed, three protocols addressing this possibility are already listed in clinicaltrials.gov (https://clinicaltrials.gov/ct2/show/NCT04332666, https://www.clinicaltrials.gov/ct2/show/NCT04401423, and https://clinicaltrials.gov/ct2/show/NCT04375124). Hence, the remarkable effects of Ang-(1-7) in the inflammatory-induced damage in the lungs and its central role in the resolution of inflammation are important factors to be considered in favor of the possibility of testing Ang-(1-7) or other Mas receptor agonists in COVID-19 patients.

## Author Contributions

GM, MR-M, DM-S, MC-S, and RS made substantial contributions to the conception and design of the work, and drafted the work. MC-S and RS revisited the manuscript critically for important intellectual content. All authors contributed to the article and approved the submitted version.

## Conflict of Interest

The authors declare that the research was conducted in the absence of any commercial or financial relationships that could be construed as a potential conflict of interest.
